# The Emotional Anatomy of the Wuhan Lockdown: Sentiment Analysis Using Weibo Data

**DOI:** 10.2196/37698

**Published:** 2022-11-14

**Authors:** Xi Chen, Michelle Yik

**Affiliations:** 1 Division of Social Science Hong Kong University of Science and Technology Hong Kong China

**Keywords:** Wuhan lockdown, COVID-19, public health emergency, emotion, circumplex model of affect, Weibo, jiayou

## Abstract

**Background:**

On January 23, 2020, the city of Wuhan, China, was sealed off in response to the COVID-19 pandemic. Studies have found that the lockdown was associated with both positive and negative emotions, although their findings are not conclusive. In these studies, emotional responses to the Wuhan lockdown were identified using lexicons based on limited emotion types.

**Objective:**

This study aims to map Chinese people’s emotional responses to the Wuhan lockdown and compare Wuhan residents’ emotions with those of people elsewhere in China by analyzing social media data from Weibo using a lexicon based on the circumplex model of affect.

**Methods:**

Social media posts on Weibo from 2 weeks before to 2 weeks after the Wuhan lockdown was imposed (January 9, 2020, to February 6, 2020) were collected. Each post was coded using a valence score and an arousal score. To map emotional trajectories during the study period, we used a data set of 359,190 posts. To compare the immediate emotional responses to the lockdown and its longer-term emotional impact on Wuhan residents (n=1236) and non-Hubei residents (n=12,714), we used a second data set of 57,685 posts for multilevel modeling analyses.

**Results:**

Most posts (248,757/359,190, 69.25%) made during the studied lockdown period indicated a pleasant mood with low arousal. A gradual increase in both valence and arousal before the lockdown was observed. The posts after the lockdown was imposed had higher valence and arousal than prelockdown posts. On the day of lockdown, the non-Hubei group had a temporarily boosted valence (*γ*_20_=0.118; SE 0.021; *P*<.001) and arousal (*γ*_30_=0.293; SE 0.022; *P*<.001). Compared with non-Hubei residents, the Wuhan group had smaller increases in valence (*γ*_21_=−0.172; SE 0.052; *P*<.001) and arousal (*γ*_31_=−0.262; SE 0.053; *P*<.001) on the day of lockdown. Weibo users’ emotional valence (*γ*_40_=0.000; SE 0.001; *P*=.71) and arousal (*γ*_40_=0.001; SE 0.001; *P*=.56) remained stable over the 2 weeks after the lockdown was imposed regardless of geographical location (valence: *γ*_41_=−0.004, SE 0.003, and *P*=.16; arousal: *γ*_41_=0.003, SE 0.003, and *P*=.26).

**Conclusions:**

During the early stages of the pandemic, most Weibo posts indicated a pleasant mood with low arousal. The overall increase in the posts’ valence and arousal after the lockdown announcement might indicate collective cohesion and mutual support in web-based communities during a public health crisis. Compared with the temporary increases in valence and arousal of non-Hubei users on the day of lockdown, Wuhan residents’ emotions were less affected by the announcement. Overall, our data suggest that Weibo users were not influenced by the lockdown measures in the 2 weeks after the lockdown announcement. Our findings offer policy makers insights into the usefulness of social connections in maintaining the psychological well-being of people affected by a lockdown.

## Introduction

### Background

The COVID-19 pandemic broke out in China in December 2019, with the city of Wuhan as its epicenter. To contain the spread of the virus, the Chinese government put Wuhan into lockdown on January 23, 2020, by imposing restrictions on travel to and from the city [1]. More rigorous lockdown measures, including limits on outdoor activities and community containment, were gradually implemented in the following weeks [2]. Although lockdowns can effectively limit the spread of a virus [3], these measures seriously interfere with citizens’ daily routines and social interactions [4]. Studies have found that travel restrictions and social distancing can lead to negative emotions such as distress, anger, and fear [5,6]. In this study, we sought to comprehensively map the emotional trajectories of Chinese people during the Wuhan lockdown using Weibo posts as our data source.

Understanding people’s emotions during public health emergencies is important. At the individual level, emotional experiences are related to mental health. Negative emotions (eg, hostility and fear) during the pandemic were found to mediate the positive correlation between exposure to stressful events (eg, experiencing a lockdown and witnessing the death of loved ones) and symptoms of anxiety and depression [7]. Positive emotions, by contrast, were positively correlated with resilience, and the correlation was stronger among people who had experienced a higher level of negative emotions in the preceding week [8,9]. High-arousal emotions (eg, anger and fear) were found to be associated with poor sleep quality, stress, and anxiety during the pandemic [10,11]. Although emotions with low arousal (eg, calmness and boredom) make people feel relaxed, they have been found to be associated with depression [12] and a loss of meaning in life [13]. People’s emotions may also contribute to behavioral outcomes in the context of public emergencies. For example, there is evidence that pleasant (eg, happiness) and unpleasant (eg, fear and anxiety) emotions were related to compliance with social distancing measures during the pandemic [14-17]. Heffner et al [18] found that pleasant emotions with higher arousal elicited by prosocial messages about COVID-19 were correlated with greater compliance with social distancing. The unrelenting and repetitive living situation was found to be associated with emotions with low arousal (eg, boredom) during the pandemic and stimulus-seeking behaviors such as impulsive buying after the pandemic [19]. Although it is clear that lockdown measures affected people’s emotions, research findings are far from conclusive.

### Why Study the Wuhan Lockdown?

Although many countries implemented lockdown measures during the pandemic [20], people’s emotional responses following the Wuhan lockdown were unique for several reasons. First, the Wuhan lockdown occurred at the beginning of the pandemic. Owing to the public’s lack of knowledge and preparation for the outbreak of a novel disease, the abrupt lockdown triggered particularly strong emotional reactions, including fear, anxiety, and hopelessness [21]. Second, the lockdown measures in China were more strictly implemented than anywhere else in the world because a wide range of local institutions (eg, residents’ committees and property management companies) were mobilized [22]. Third, the Wuhan lockdown was implemented just before the Chinese New Year, a major holiday in Chinese communities. The mobility restrictions prevented individuals from participating in significant activities such as family reunions, leading to intense emotional responses (eg, anxiety, disappointment, anger, and hostility) and worsened psychological well-being [2,4,23,24].

Studying the emotional responses of Wuhan residents can be important as those who were physically restricted by the lockdown measures were more emotionally vulnerable to pandemic-related events. Studies have found that residents of Wuhan and the rest of the Hubei province were sensitive to milestone events (eg, the lockdown announcement on January 23, 2020, and the death of Dr Li Wenliang on February 7, 2020) and expressed more negative emotions after the lockdown was imposed than people from elsewhere in China [2,4,25,26]. In this study, we used sentiment analysis of Weibo data and the circumplex model of affect to examine the emotional responses of Chinese people in general and those living in Wuhan in particular.

### Why Examine the Wuhan Lockdown Using Social Media Posts?

During the pandemic, social media became a major source of information about the public’s emotions in response to lockdowns [27]. Unlike surveys, which rely on retrospective self-reports, social media data include real-time spontaneous feelings from a wide variety of people and are free from recall bias and demand characteristics [25,28,29]. Starting from the outbreak of COVID-19, a massive wave of discussions took place on Sina Weibo, one of China’s most popular social media platforms [2,27]. In March 2022, Weibo had 582 million active users per month and 252 million active users per day [30]. Emotion information embedded in posts on Weibo provides a unique source of data for researchers to investigate emotions in response to the Wuhan lockdown. Therefore, in this study, we used Weibo posts to map the emotional trajectories of Chinese people before and after the lockdown was imposed.

Using social media data, recent studies have found an increase in negative emotions such as fear, panic, guilt, anger, and disappointment [2,4,25,26,28,31] as well as an increase in positive emotions such as feeling blessedness, hope, admiration, and encouragement after the Wuhan lockdown [2,26,32]. It appears that Chinese people experienced increases in both pleasant and unpleasant emotions after the lockdown was imposed. However, researchers have used several discrete emotion categories to code social media data. For example, Su et al [26] used the negative emotion and affect categories from the Simplified Chinese Linguistic Inquiry and Word Count lexicon [31]. Su et al [28] and Yu et al [25] adopted “depression,” “like,” “dislike,” “anger,” “sadness,” “fear,” “enjoyment,” “disgust,” and “surprise” from the Affective Lexicon Ontology [33]. Shen et al [2] manually coded emotion words from corpora as “anger,” “fear,” “neutral,” “encouragement,” and “hope.” Cao et al [32] used the Ortony, Clore, and Collins model and the 6 emotions by Ekman to categorize emotions as “joy,” “hope,” “distress,” “fear,” “admiration,” “reproach,” and “neutral.” The limited emotion types used in previous studies might not be sufficient to construct a complete picture of emotional responses during the Wuhan lockdown. Applying a sentiment analysis based on the circumplex model of affect, this study sought to map the comprehensive emotion trajectories of Chinese people during the Wuhan lockdown.

### The Circumplex Model of Affect

The circumplex model of affect defines emotion in a Cartesian space with 2 independent dimensions and has received support in various cultural settings [34-37]. The horizontal axis, valence, denotes the degree of pleasantness and unpleasantness, and the vertical axis, arousal, denotes the degree of activation and deactivation. An emotion is composed of different levels of valence and arousal. For instance, although both *calmness* and *encouragement* are pleasant emotions, the former is less activated than the latter. Compared with categorical models in which emotions are restricted to a certain number of categories, the circumplex model allows for a potentially infinite number of emotions, thus enabling a more fine-grained analysis of emotion in sentiment analysis [38].

Although previous research findings have shown that both pleasant and unpleasant emotions were related to compliance with social distancing during the pandemic, the arousal dimension of the circumplex model of affect has been largely overlooked. For example, Shen et al [2] sorted emotions into “positive,” “negative,” and “neutral” categories, neglecting the arousal dimension. Researchers have mainly focused on high-arousal unpleasant emotions such as fear, panic, and anger [28] while neglecting low-arousal emotions such as calmness and glumness [36]. To obtain a full picture of the emotional trajectories of Chinese people in response to the Wuhan lockdown, we used a lexicon based on the circumplex model of affect for sentiment analysis in this study.

### Objectives

Understanding citizens’ emotional responses to extreme social distancing measures such as a lockdown provides insights into how governments can effectively respond to a pandemic. The Wuhan lockdown certainly served as a catalyst for research on mapping the emotions of those affected by lockdown measures, but the findings remain far from conclusive. The aim of this study was to examine the emotional trajectories expressed on Weibo using a sentiment analysis approach. Specifically, we sought to answer three research questions (RQs): (1) What were the emotional trajectories of Chinese people during the Wuhan lockdown? (RQ 1); (2) Were there any differences in immediate emotional responses to the lockdown between people from Wuhan and those from non-Hubei areas? (RQ 2); and (3) Were there any differences in the longer-term impact of the lockdown on emotional responses between people from Wuhan and those from non-Hubei areas? (RQ 3).

## Methods

### Data Sets

#### Overview

To explore the emotional responses of Chinese people, we extracted 2 data sets from Weibo, each using a different set of keywords. From both data sets, we extracted posts created from 2 weeks before to 2 weeks after the lockdown announcement (ie, January 9, 2020, to February 6, 2020) and removed posts that yielded missing valence and arousal values. When the posts contained words not coded in the lexicon, missing values were assigned.

We included the 2 weeks before the lockdown in the analysis to create a full picture of prelockdown emotional trajectories. The limit of 2 weeks after the lockdown announcement was chosen to avoid the potential influence of the death of whistleblower Dr Li Wenliang on February 7, 2020 [25]. This event was accompanied by a dramatic increase in the number of posts and content characterized by negative emotions such as anger, fear, surprise, and depression [4,25,27], which could bias the emotional trajectories immediately following the lockdown announcement.

#### Data Set 1

The data in data set 1 were collected using Weibo’s built-in advanced search tool, through which the search can be optimized by specifying keywords, time frames, and categories (eg, original posts or reposts). A crawler was developed using the *httr* and *rvest* packages in R (R Foundation for Statistical Computing) [39,40]. The keyword “武汉” (“Wuhan”) was used in the search. The data set was created in June 2020 and contained 602,737 original posts made by 396,054 users from January 1, 2020 to February 15, 2020. After removing posts with missing values and extracting posts created from 2 weeks before to 2 weeks after the lockdown announcement, the final data set included 359,190 posts by 242,023 users.

#### Data Set 2

The data in data set 2 were extracted from a publicly available data set named Weibo-COV2 that contains >65 million Weibo posts made by 20 million active users from December 2019 to December 2020 [41]. The authors of Weibo-COV2 defined active users as those with >50 followers and 50 fans who had made a post within the 30 days before the data were collected. Inactive users were excluded as they did not make any posts during the pandemic. The authors first built a pool of active users whose posts were screened using 492 pandemic-related keywords. The 492 keywords used by the authors [41] covered events (eg, “武汉封城,” implying “Wuhan lockdown”), drugs and supplies (eg, “口罩,” implying “masks”), experts and physicians (eg, “钟南山,” implying “Zhong Nanshan”), and government policies (eg, “延迟开学,” implying “postponing school reopening”). In this study, we extracted Weibo-COV2 data from 2 weeks before to 2 weeks after the Wuhan lockdown announcement and removed posts with missing valence and arousal values. As we aimed to explore individuals’ emotional responses before and after the lockdown was imposed, data from users who created only 1 post were excluded. Ultimately, data set 2 contained 974,317 posts—282,442 (28.99%) made before the lockdown announcement and 691,875 (71.01%) made after the lockdown announcement—by 219,446 unique users, each of whom made at least one post before and one post after the lockdown was imposed.

#### Comparing the 2 Data Sets

Each of our 2 data sets had strengths and limitations. Together, they provided an effective platform to address our 3 RQs.

First, different keywords were used to create each data set. The keyword “Wuhan” for data set 1 allowed us to understand the daily emotional trajectories before pandemic-related topics went viral on Weibo, providing a baseline measure. By contrast, the pandemic-related keywords for data set 2 allowed us to explore people’s emotions regarding the pandemic. On January 20, 2020, when human-to-human transmission was confirmed, the number of daily posts on Weibo-COV2 increased dramatically [27,41]. To comprehensively map the emotional trajectory of Chinese people during the lockdown period, it is necessary to use both data sets 1 and 2.

Second, the 2 data sets had different structures. The data in data set 1 were cross-sectional in nature and included posts made by different people over time. Most user IDs (208,389/242,023, 86.1%) had only 1 corresponding post. Such cross-sectional data provide snapshots of Chinese people’s emotions at different time points. By contrast, as data set 2 included all pandemic-related posts from each user, we were able to map their emotions before, during, and after the lockdown was imposed.

We used data set 1 to explore emotional trajectories before and after the Wuhan lockdown was imposed (RQ 1). Using data set 2, we compared the immediate emotional responses to the lockdown (RQ 2) and its longer-term emotional impact (RQ 3) for people from Wuhan and non-Hubei people.

### Ethical Considerations

To protect human participants’ privacy, all users’ personal information was anonymized during our analysis. This study focused on human emotions at the aggregated level, meaning that individual-level information was averaged across users and no individual information was disclosed.

### Data Cleaning

**Figure 1:**
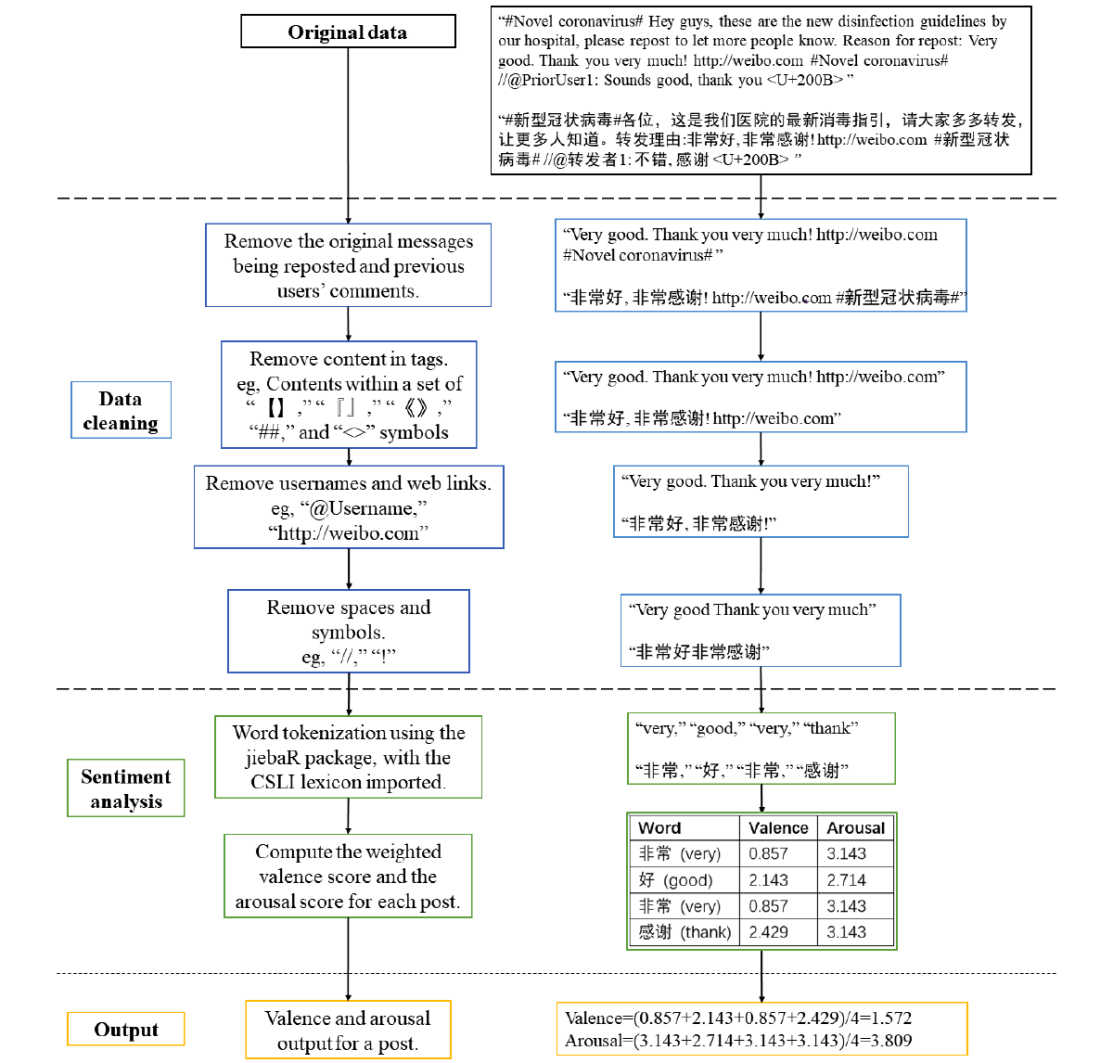
Process of data cleaning and sentiment analysis with post examples. CSLI: Chinese Sentiment Lexicon for Internet.

We cleaned the posts before assigning emotion scores. Figure 1 summarizes the process of data cleaning and sentiment analysis. Weibo posts have two major formats: original posts and reposts. An original post contains the original comments made by a user. A repost usually has three parts: the original message being reposted, other users’ comments about the original post, and the reposting user’s comments about the original post. As previous studies found that including the message being reposted in sentiment analysis significantly reduced the unpleasantness of emotions in Weibo posts, we excluded the original message being reposted and previous users’ comments and analyzed the comments by the reposting user only [42]. For example, a repost might read, “Hey guys, these are the new disinfection guidelines by our hospital, please repost to let more people know. Reason for repost: Very good. Thank you very much! //@PriorUser1: Sounds good, thank you.” In this repost, only the phrase “Very good. Thank you very much!” is an original comment made by the reposting user and would be subjected to our sentiment analysis. However, if the reposting user added no comments, we conducted sentiment analysis on all previous users’ comments to represent the reposting user’s comment as previous studies have found that Weibo users only repost other users’ comments when they agree with them [43,44].

During data cleaning, we first retained the original posts and the comments made by the reposting user. We then removed tags, usernames, and web links from each post as such content would add noise to the analysis. The remaining content of each post was retained for sentiment analysis.

### Sentiment Analysis

A lexicon-based sentiment analysis was conducted to assign each post a valence score and an arousal score based on the Chinese Sentiment Lexicon for Internet (CSLI) [45], which consists of 7088 Chinese words, each of which is annotated with a valence score ranging from −4 to 4 and an arousal score ranging from 0 to 8.

Our sentiment analysis procedure is summarized in Figure 1. The *JiebaR* package in R [46] was used for tokenization, which involved extracting the emotion words listed in the CSLI from each post. Following the procedures by Zhao et al [45], we calculated the valence and arousal scores of each post using the weighted average value. For each post, the valence score of each emotion word was multiplied by its frequency—the products of all emotion words in a post were summed and then divided by the total frequency of all emotion words in the post. The same method was applied to calculate the arousal score. Mean valence and arousal scores were assigned to each post to indicate its emotion. Using Weibo posts, Zhao et al [45] validated this sentiment analysis method by comparing valence and arousal scores rated by participants with the predicted CSLI scores for the same posts. The convergence was 0.70 for valence and 0.59 for arousal. As valence in the CSLI lexicon has a range of −4 to 4, we defined posts with valence scores <0 as *unpleasant* and scores >0 as *pleasant*. Similarly, as arousal has a range of 0 to 8, we defined posts with arousal scores <4 as *low-arousal* and scores >4 as *high-arousal*.

For subsequent analyses, we computed 2 types of daily emotion scores using the valence and arousal scores of posts yielded by the CSLI lexicon. One was daily emotion by person. For example, a daily valence score by person was calculated for a user by averaging the valence scores of all posts made by the user on that day. A daily arousal score by person was calculated in the same way. The other type of daily emotion score was emotion by day. A valence score by day was calculated by averaging all daily valence scores by person across users who created posts on a particular day. An arousal score by day was calculated in the same way.

### Geographical Location: Wuhan Versus Non-Hubei Residents

People who were physically restricted by the lockdown measures might have had different emotional responses from people who were not in the lockdown area. A previous study found that Hubei residents expressed more negative emotions than those living elsewhere in China during the pandemic [2]. To examine the differences in emotions in response to the Wuhan lockdown between Wuhan and non-Hubei areas, we grouped the Weibo users in data set 2 into a Wuhan group (ie, the lockdown area) and a non-Hubei group (ie, nonlockdown area) based on their postlockdown geographical location. The non-Hubei area, rather than the non-Wuhan area, was used as Wuhan was not the only city in the Hubei province that was locked down after January 23, 2020. A total of 15 other cities in Hubei gradually implemented similar restrictions up to January 27, 2020 [47]. Therefore, to compare the pre– and post–lockdown announcement periods, we decided to focus on the non-Hubei rather than the non-Wuhan area.

To differentiate between Wuhan and non-Hubei users, the IDs of users who made posts after the announcement that contained geotags (ie, the GPS coordinates of a user’s device, including longitude and latitude) were filtered for geographical information [48]. As people could no longer enter or exit Wuhan after the lockdown was imposed, the users’ GPS information enabled us to assess their actual geographical locations after the lockdown was imposed.

We used data set 2 to explore the emotional responses of the Wuhan and non-Hubei groups. We first used the geotags to separate user IDs into Wuhan and non-Hubei groups. All posts with these user IDs were then extracted, resulting in 6665 posts generated by 1236 users in Wuhan and 51,020 posts generated by 12,714 users outside Hubei. These users all made at least one post before and one post after the lockdown was imposed. It is important to note that Weibo users can select geotags from a list of locations, allowing them to misreport their actual locations. This function is an unfortunate drawback of Weibo data and should be treated as a source of error [48].

### Multilevel Modeling

We used data set 2 to examine how people’s everyday emotions evolved from 2 weeks before to 2 weeks after the lockdown was imposed. As data set 2 had a nested data structure in which repeated measures of emotion ratings were nested within each user, we used multilevel modeling [49]. A multilevel model separates the residual variances of a sample into within-person (level 1) and between-person (level 2) variances [50]. Multilevel modeling allows for data dependency [51,52], meaning that the measures of one person would be more similar to one another than to the measures of another person. In addition, social media data are not always complete, meaning that there would be missing observations if an individual did not post anything on certain days. Multilevel modeling is suitable for social media data as it can tolerate missing observations [51].

Piecewise multilevel models can capture changes in groups during different phases [53] and can therefore be used to compare the changes in emotional trajectories between the Wuhan and non-Hubei groups before and after the lockdown was imposed. The number of posts in data set 2 was small before January 20, 2020, as widespread discussion of pandemic-related topics had not yet started [27,41]. To ensure adequate observations of emotion ratings across users per day (ie, >50 users per day) [54], we used data set 2 data starting from January 20, 2020, for the multilevel modeling. The R package *lme4* [55] was used to estimate the multilevel models.

## Results

### Emotion Map

**Figure 2:**
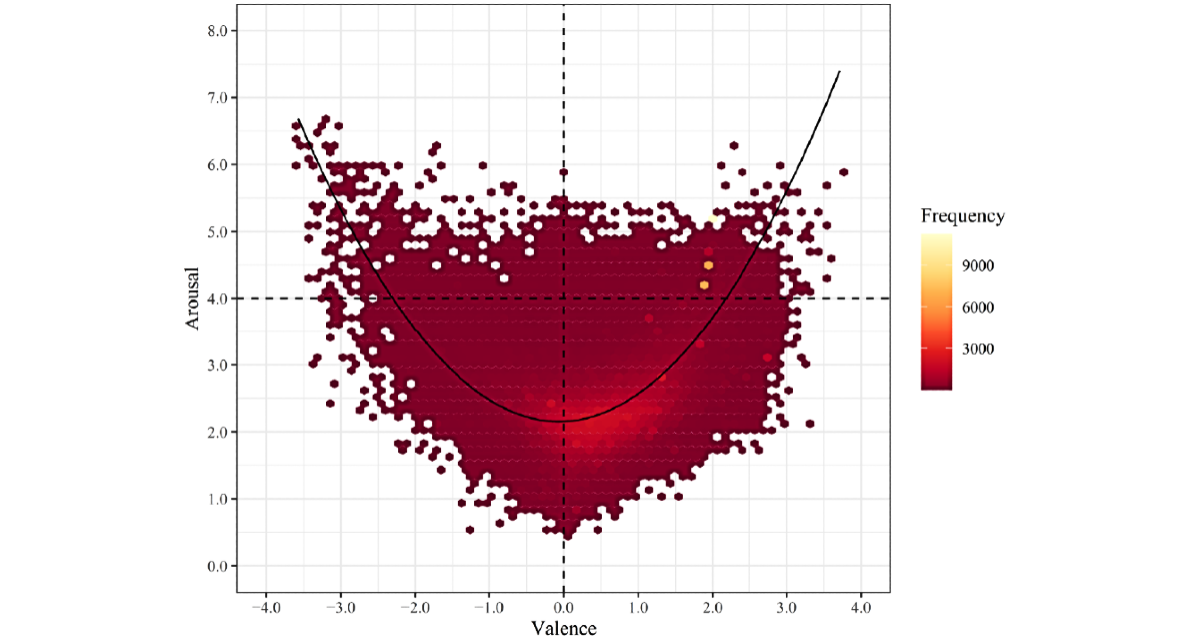
Emotion map of 359,190 Weibo posts by 242,023 users from January 9, 2020, to February 6, 2020 (data set 1).

The data in data set 1 included 359,190 posts made by 242,023 users. To map the emotion distribution of Weibo posts, the valence and arousal scores of each post were used to draw the scatterplot shown in Figure 2. The valence and arousal combinations are depicted by their frequency. Posts were classified as *unpleasant* when their valence scores were <0 and as *pleasant* when their valence scores were >0. Posts were classified as *low-arousal* when their arousal scores were <4 and as *high-arousal* when their arousal scores were >4.

Overall, the emotions in the posts spread across the valence-arousal plane, covering both the pleasant and unpleasant areas. Highly frequent combinations of valence and arousal scores were gathered in low-arousal areas and leaned toward the pleasant half of the plane. Several highly frequent valence-arousal combinations were in the pleasant and high-arousal quadrants (valence >0; arousal >4).

Of the 359,190 posts, 248,757 (69.25%) fell in the pleasant and low-arousal quadrant (valence >0; arousal <4), 72,773 (20.26%) fell in the unpleasant and low-arousal quadrant (valence <0; arousal <4), 32,850 (9.15%) fell in the pleasant and high-arousal quadrant (valence >0; arousal >4), and 2023 (0.56%) fell in the unpleasant and high-arousal quadrant (valence <0; arousal >4). The remaining 0.78% (2787/359,190) of posts were neutral in valence or had a moderate arousal of 4. These results showed that, among all the combinations of valence and arousal, most posts (282,068/359,190, 78.53%) made from January 9, 2020, to February 6, 2020, were pleasant, among which there were more low- than high-arousal emotions.

To evaluate the relationship between the valence and arousal of posts on Weibo, a quadratic regression was fitted to the 359,190 posts. The results showed a significant quadratic relationship between valence and arousal (*F*_2,359,187_=1.554×10^5^; *P*<.001; *R*^2^=0.464). The regression equation was arousal = 2.156 + 0.046 × valence + 0.368 × valence^2^. As shown in Figure 2, both pleasant and unpleasant emotions were accompanied by activation (refer to the study by Yik et al [37]).

### Daily Emotional Trajectories Before and After the Lockdown Was Imposed

**Figure 3:**
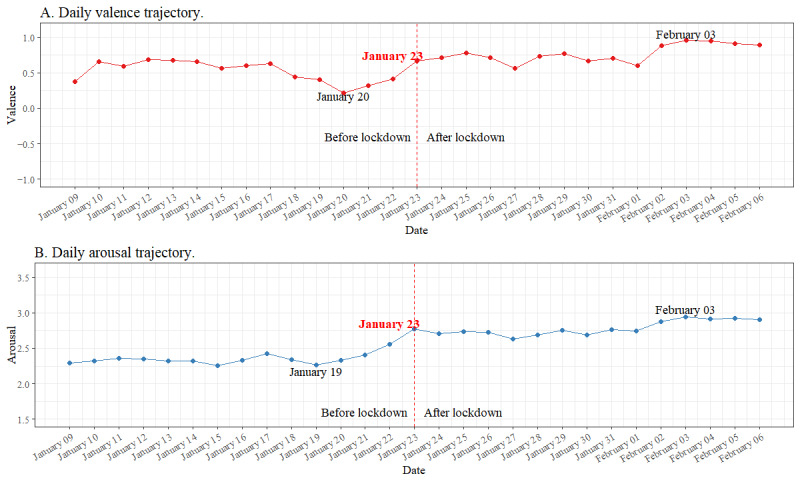
Cross-sectional emotion trajectories from January 9, 2020, to February 6, 2020 (data set 1).

To explore the emotional trajectories before and after the lockdown was imposed (RQ 1), the data in data set 1 were analyzed. We averaged the valence of all posts made on each day and plotted the daily valence scores over the 4 weeks. Figure 3A displays the daily trajectories in valence (−4 to +4). On January 20, 2020, when human-to-human transmission of COVID-19 was confirmed [25], a turning point emerged during which the valence score increased until January 23, 2020, and then reached a plateau and stabilized at a higher level. On February 3, 2020, a day after all patients infected or suspected to be infected with SARS-CoV-2 were required to be quarantined and isolated [25], valence rose to a higher level than the week after the lockdown announcement, reaching a second plateau. The results show that the emotions expressed on Weibo became more positive starting on January 20, 2020, 3 days before the lockdown. After the lockdown was imposed, a higher level of pleasant emotions was expressed, although valence was generally pleasant (valence >0) at a low level (arousal <4). Two valence plateaus were observed: one from January 23, 2020, to February 2, 2020, and one from February 3 to 6, 2020.

Similarly, we averaged the arousal of all posts made on each day and plotted the daily arousal scores over the 4 weeks. Figure 3B displays the daily trajectory of arousal scores (0 to 8). The plot shows that the turning point of arousal occurred on January 19, 2020, after which arousal increased until January 23, 2020, and then reached a first plateau and became stable. From February 3, 2020, arousal rose to a higher level than the week after the lockdown was imposed and reached a second plateau. These results show that the emotions expressed by Weibo users became more activated during the lockdown, although the daily arousal level was generally low. Two arousal plateaus were observed: one from January 23, 2020, to February 2, 2020, and one from February 3 to 6, 2020.

**Figure 4:**
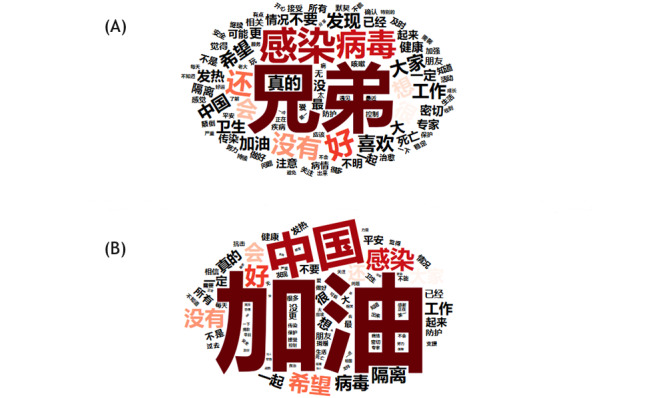
Word cloud of the (A) 100 most frequently used words in the 2 weeks before the lockdown and the (B) 100 most frequently used words in the 2 weeks after the lockdown was imposed (data set 1).

To investigate the reasons for the 2 valence and arousal plateaus during the lockdown, we conducted separate lexical analyses on the word frequencies across all words that were used during the 2 plateau periods. Word clouds, in which more frequently used words appear in larger font sizes, were used to visualize word frequency and demonstrate the main themes of the posts [56]. The 100 most frequently used words in the 2 weeks before the lockdown (January 9, 2020, to January 22, 2020) are illustrated in Figure 4A. The word *兄弟* (*brothers*) was the most commonly used word in the 2 weeks before the lockdown as fans of a music band named *摩登兄弟* (*Modern Brothers*) were doing promotion activities. This result provides a picture of Weibo topics before the lockdown, during which people on Weibo engaged in celebrity promotion activities and discussed topics other than the COVID-19 pandemic [57,58].

As shown in Figure 4B, of the 100 most frequently used words in the 2 weeks after the lockdown announcement (January 23, 2020, to February 6, 2020), *加油* (*jiayou* or *add oil*, which is a term of encouragement) had the highest frequency, accounting for 4.32% (81,969/1,895,476) of all emotion words found in the posts. The prevalence of *jiayou* increased to 6.48% (57,239/883,447) from February 3 to 6, 2020. Notably, *jiayou* accounted for only 0.53% (6734/1,277,449) of emotion words in the 2 weeks before the lockdown. In the CSLI lexicon, *jiayou* has a valence score of 2.000 (ranging from −4 to +4) and an arousal score of 5.143 (ranging from 0 to 8). It is likely that the escalating valence of Weibo posts during the lockdown can be attributed to an increased use of words of encouragement such as *jiayou*.

### The Emotional Trajectories of Wuhan and Non-Hubei Residents

**Figure 5:**
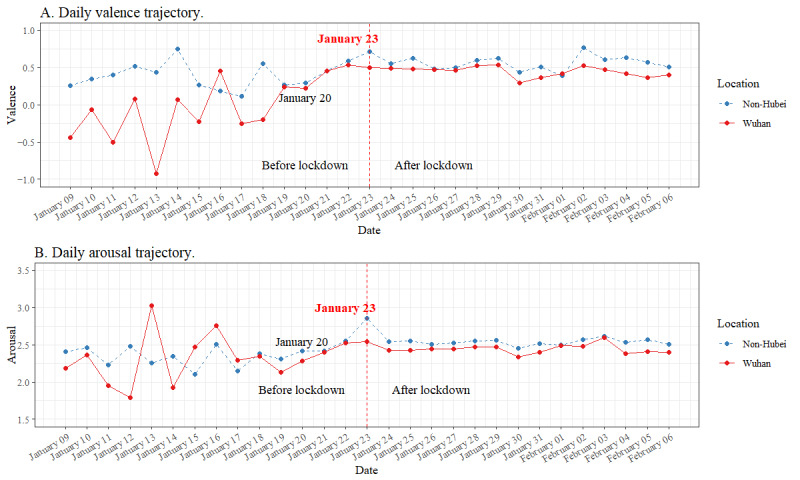
Daily valence and arousal trajectories in the Wuhan and non-Hubei groups from January 9, 2020, to February 6, 2020 (data set 2).

Data set 2 included 6665 posts made by 1236 Wuhan residents and 51,020 posts made by 12,714 non-Hubei residents from 2 weeks before to 2 weeks after the lockdown announcement. Each user made at least one post before and one post after the announcement. To examine the daily valence trajectories of Wuhan and non-Hubei residents, we averaged the daily valence across users on each day to generate daily valence scores for the Wuhan and non-Hubei groups. The daily arousal trajectories of Wuhan and non-Hubei residents were generated using the same method. Figure 5A shows the daily valence trajectories of the Wuhan group and the non-Hubei group during the same period. Before human-to-human transmission of the virus was confirmed on January 20, 2020, the valence of the Wuhan group fluctuated more dramatically than that of the non-Hubei group, possibly because the data points were dominated by a small amount of data across individuals per day. Before January 20, 2020, the number of people who created posts per day in the Wuhan group ranged from 1 to 45, whereas the number of people who created posts per day in the non-Hubei group ranged from 11 to 91.

From January 20, 2020, the number of people who made posts per day dramatically increased in both the Wuhan and non-Hubei groups to a minimum of 162 individuals per day, which is consistent with the findings of Lu et al [27] and Hu et al [41], who reported a dramatic increase in Weibo-COV2 data after January 20, 2020. From January 20, 2020, the daily valence of the Wuhan and non-Hubei groups became parallel and increased steadily until the lockdown announcement on January 23, 2020, when the valence of the non-Hubei group peaked but that of the Wuhan group remained at a similar level to that of the previous day. After January 23, 2020, the daily valence of both groups increased and became similar across days. Notably, unlike the non-Hubei group, the Wuhan group did not express more pleasant emotions on the day of lockdown.

Figure 5B shows a plot of daily arousal trajectories of the Wuhan and non-Hubei groups during the same period. The trajectories of both groups became parallel and increased after January 20, 2020, before diverging on January 23, 2020, when arousal peaked in the non-Hubei group but remained steady in the Wuhan group. After the lockdown was imposed on January 23, 2020, the daily arousal trajectories of both groups stabilized at a higher level and became parallel across days. Unlike the non-Hubei group, the Wuhan group did not express more activated emotions on the day of lockdown.

### Immediate Emotional Responses of Wuhan and Non-Hubei Weibo Users to the Wuhan Lockdown

#### Preliminary Analysis

To compare the immediate emotional responses of Wuhan and non-Hubei residents to the lockdown (RQ 2), multilevel modeling was conducted using data set 2. The descriptive statistics are provided in Table 1. The skewness of the valence scores ranged from −0.188 to 1.633, and the kurtosis ranged from 0.892 to 3.982, both of which are within the acceptable range for data normality (−2 to +2 for skewness and −7 to +7 for kurtosis) [59,60]. The skewness of the arousal scores ranged from 1.393 to 1.633, and the kurtosis ranged from 2.499 to 3.982, both of which are also within the acceptable range for data normality.

**Table 1:** Descriptive statistics of valence and arousal by person by day for the Wuhan and non-Hubei groups (data set 2).

Emotional response and group			Users, N		Mean (SD)		Skew		Kurtosis
**Valence (−4 to +4)**									
	Non-Hubei	12,714		0.555 (0.913)		−0.188		0.892	
	Wuhan	1236		0.453 (0.845)		0.004		1.220	
**Arousal (0 to 8)**									
	Non-Hubei	12,714		2.544 (0.815)		1.393		2.499	
	Wuhan	1236		2.446 (0.718)		1.633		3.982	

#### Multilevel Modeling

To examine the proportion of variance of valence accounted for by between- and within-person levels, we first used an empty multilevel model with random intercept only [51]. The results indicated a significant between-person variance of 0.332 (*χ*^2^_4_=1187.2; *P*<.001). The intraclass correlation coefficient was 0.134, implying that the between-person variance accounted for 13.40% of the variance. The same analysis was repeated for the arousal scores. The results indicated a significant between-person variance of 0.252 (*χ*^2^_4_=699.6; *P*<.001). The intraclass correlation coefficient was 0.098, implying that the between-person variance in arousal accounted for 9.77% of the variance. These results justified multilevel analysis at both level 1 (within-person) and level 2 (between-person).

**Figure 6:**
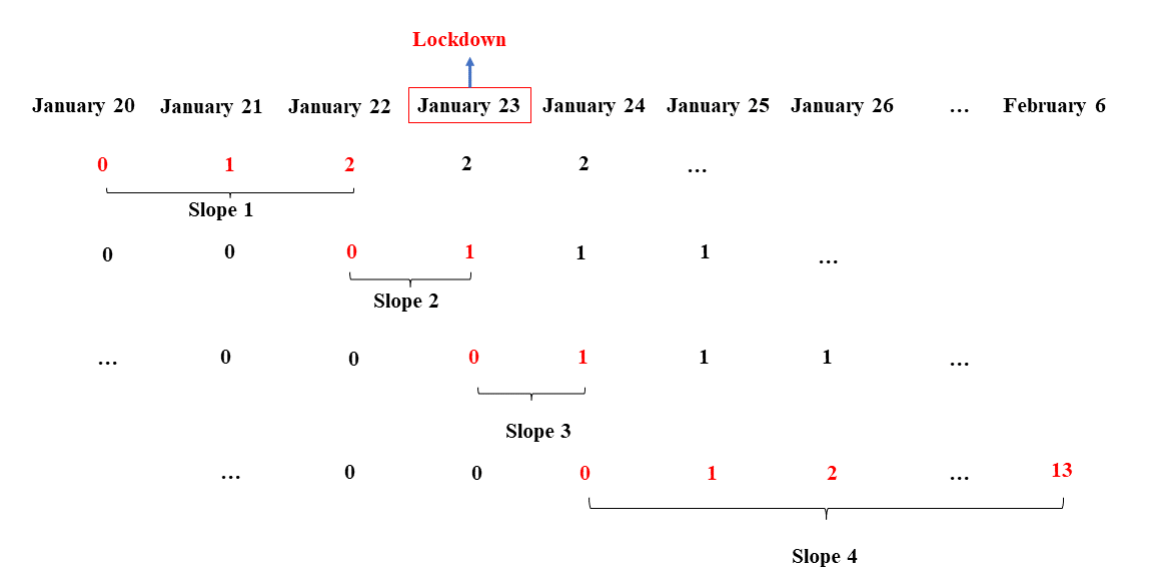
Coding scheme for slopes 1 to 4 in multilevel modeling.

To compare the emotional responses to the lockdown of Wuhan and non-Hubei residents (RQ 2), we introduced 4 slopes into the multilevel model. To ensure adequate observations of emotion ratings across users per day (ie, >50 users per day) [54], we used data after January 20, 2020, in data set 2. Slope 1 modeled the emotional trajectories between January 20, 2020, and January 22, 2020, just before the lockdown. To compare the immediate emotional responses to the lockdown of Wuhan and non-Hubei residents, slopes 2 and 3 were introduced into the model. Slope 2 tested whether the emotions on the day of lockdown (January 23, 2020) were different from the emotions on the day before (January 22, 2020); slope 3 tested whether the emotions on the day of lockdown (January 23, 2020) were different from the emotions the following day (January 24, 2020). To compare the longer-term emotional impact on Wuhan and non-Hubei residents after the lockdown was imposed, we used slope 4 to model emotional changes in the 2 weeks after the lockdown was imposed (ie, between January 24, 2020, and February 6, 2020). Figure 6 summarizes the coding scheme for the 4 slopes. The dates on or before the start date of a slope were coded as 0, and the dates after the start date of a slope were coded with a sequential integer depending on the distance from the start date. For example, as slope 4 started on January 24, 2020, the dates on or after January 24 were coded as 1 for January 25, 2 for January 26, and so on.

The outcomes of all models were tested using the daily emotion data (ie, either valence or arousal) of each person. Whenever location was included as a between-person (level 2) predictor, it was dummy coded as 1, denoting the Wuhan group, or 0, denoting the non-Hubei group.

The random effects of the slopes were introduced into the models based on the RQs. To compare the immediate emotional responses to the lockdown of the Wuhan and non-Hubei groups from the day before to the day after the lockdown was imposed, we estimated the random effects (ie, variability across users in Wuhan and non-Hubei groups) of slope 2 (the emotional trend from January 22, 2020, to January 23, 2020) and slope 3 (the emotional trend from January 23, 2020, to January 24, 2020). Location was introduced as the level-2 predictor. In this model, we examined the fixed effects of slope 1 (January 20, 2020, to January 22, 2020) and slope 4 (2 weeks after the lockdown was imposed) to test the overall changes in both the Wuhan and non-Hubei groups. In all the models, *i* denoted an individual, and *t* denoted a date. Multilevel model 1 is shown in Textbox 1.

**Multilevel model 1 for immediate emotional responses.**

**Multilevel model 1**

- Level 1:
  Emotion_it_ = *β*_0i_ + *β*_1i_ × slope 1 + *β*_2i_ × slope 2 + *β*_3i_ × slope 3 + *β*_4i_ × slope 4 + e_it_
- Level 2:
  *β*_0i_ = *γ*_00_ + *γ*_01_ × location + U_0i_
  *β*_1i_ = *γ*_10_
  *β*_2i_ = *γ*_20_ + *γ*_21_ × location + U_2i_
  *β*_3i_ = *γ*_30_ + *γ*_31_ × location + U_3i_
  *β*_4i_ = *γ*_40_

#### Valence

The results of the multilevel modeling with valence as the outcome variable are summarized in the upper panel of Table 2. Before the lockdown, the overall valence of Wuhan and non-Hubei residents yielded a positive coefficient of 0.123, implying an increase in average valence between January 20, 2020, and January 22, 2020 (SE 0.013; *P*<.001).

As location was introduced as a moderator for slopes 2 and 3 in multilevel model 1, the main effects of slopes 2 and 3 indicated the emotion trends conditional at a location of 0 (ie, the non-Hubei group). To test the emotional changes in response to the lockdown, we relied on slope 2. The main effect of slope 2 was positive (*γ*_20_=0.118; SE 0.021; *P*<.001), implying that the valence score of the non-Hubei group increased from the day before. The random effect of slope 2 was not significant (SD 0.259; *χ*^2^_3_=3.8; *P*=.28), implying that there were no significant variations in slope 2 across individuals in either the Wuhan or non-Hubei group. However, the interaction between slope 2 and location yielded a significant result of −0.172 (SE 0.052; *P*=.001). This significant moderation of location with an insignificant random effect can be interpreted as a systematic difference between the Wuhan and the non-Hubei groups [53]. Although there was no difference in the valence trend of the individuals in the Wuhan and non-Hubei groups, the mean valence increase was higher in the non-Hubei group than in the Wuhan group. In other words, the non-Hubei residents reacted to the lockdown more positively than the Wuhan residents.

To test the emotional change immediately after the lockdown was imposed, we relied on slope 3. The main effect of slope 3 was negative (*γ*_30_=−0.146; SE 0.020; *P*<.001), indicating that the valence score of the non-Hubei residents decreased after the day of lockdown. The random effect of slope 3 was significant (SD 0.260; *χ*^2^_3_=24.7; *P*<.001), implying that the slope varied across individuals in both the Wuhan and non-Hubei groups. Location significantly moderated slope 3 and, therefore, the valence score of the Wuhan users did not decrease as much as that of the non-Hubei users (*γ*_31_=0.109; SE 0.047; *P*=.02). In other words, the valence score of the non-Hubei residents decreased more than that of the Wuhan residents.

**Table 2:** Immediate emotional responses based on a multilevel model predicting valence or arousal with slope 2 and slope 3 at level 1 by location at level 2 (data set 2).

Variable		Fixed effect				Random effect		
		Coefficient	SE	*P* value	SD		Chi-square (*df*)^a^	*P* value
**Valence**								
	Intercept (*γ*_00_)	0.337	0.022	<.001	0.452		656.8 (3)	<.001
	Slope 1 (*γ*_10_)^b^	0.123	0.013	<.001	—^c^		—	—
	Slope 2 (*γ*_20_)^d^	0.118	0.021	<.001	0.259		3.8 (3)	.28
	Slope 3 (*γ*_30_)^e^	−0.146	0.020	<.001	0.260		24.7 (3)	<.001
	Slope 4 (*γ*_30_)^f^	0.000	0.001	.99	—		—	—
	Location (*γ*_01_)^g^	−0.037	0.032	.24	—		—	—
	Slope 2×location (*γ*_21_)^d,g^	−0.172	0.052	.001	—		—	—
	Slope 3×location (*γ*_31_)^e,g^	0.109	0.047	.02	—		—	—
**Arousal**								
	Intercept (*γ*_00_)	2.365	0.019	<.001	0.265		183.3 (3)	<.001
	Slope 1 (*γ*_10_)^b^	0.089	0.011	<.001	—		—	—
	Slope 2 (*γ*_20_)^d^	0.293	0.022	<.001	0.569		333.2 (3)	<.001
	Slope 3 (*γ*_30_)^e^	−0.315	0.021	<.001	0.607		403.4 (3)	<.001
	Slope 4 (*γ*_40_)^f^	0.000	0.001	.30	—		—	—
	Location (*γ*_01_)^g^	−0.042	0.026	.11	—		—	—
	Slope 2×location (*γ*_21_)^d,g^	−0.262	0.053	<.001	—		—	—
	Slope 3×location (*γ*_31_)^e,g^	0.218	0.050	<.001	—		—	—

^a^The chi-square value is calculated as the difference in –2loglikelihood between a model with and a model without the random effect.

^b^Slope 1: emotional trend before the lockdown (between January 20, 2020, and January 22, 2020).

^c^The cells were kept empty when the random effect of a variable was not included in the model.

^d^Slope 2: difference in emotion between January 23, 2020 (the day of lockdown) and January 22, 2020.

^e^Slope 3: difference in emotion between January 23, 2020 (the day of lockdown) and January 24, 2020.

^f^Slope 4: longer-term emotional trend in the 2 weeks after the lockdown was imposed.

^g^Location: dummy coded as 1 denoting the Wuhan group and 0 denoting the non-Hubei group.

#### Arousal

The results of the multilevel modeling with arousal as the outcome variable are summarized in the lower panel of Table 2. From January 20, 2020, to January 22, 2020, the overall arousal trend of the Wuhan and non-Hubei groups yielded a positive coefficient (*γ*_10_=0.089; SE 0.011; *P*<.001), indicating an increase in average arousal.

The main effect of slope 2 was positive (*γ*_20_=0.293; SE 0.022; *P*<.001), indicating that the arousal score of the non-Hubei group on the day of lockdown increased from the day before. The random effect of slope 2 was significant (SD 0.569; *χ*^2^_3_=333.2; *P*<.001), implying that slope 2 varied across individuals in both the Wuhan and non-Hubei groups. Location significantly moderated slope 2 (*γ*_21_=−0.262; SE 0.053; *P*<.001), indicating that the non-Hubei group reacted to the lockdown with a greater increase in arousal than that of the Wuhan users.

The main effect of slope 3 had a coefficient of −0.315 (SE 0.021; *P*<.001), indicating that the arousal score of the non-Hubei residents decreased after the day of lockdown. The random effect of slope 3 was significant (SD 0.607; *χ*^2^_3_=403.4; *P*<.001), indicating that the slope varied across individuals in both the Wuhan and non-Hubei groups. Location significantly moderated slope 3 (*γ*_31_=0.218; SE 0.050; *P*<.001), indicating that the non-Hubei group reacted to the lockdown with a greater drop in arousal than that of the Wuhan users.

Taken together, the results indicate that the non-Hubei users’ emotions were influenced by the Wuhan lockdown and became more pleasant and aroused. However, the effect was temporary. Both the valence and arousal of the non-Hubei group decreased immediately after the day of lockdown, and the magnitude of the decreases in the non-Hubei group was significantly greater than that in the Wuhan group. In other words, the emotions of Wuhan residents were less affected by the lockdown.

### Longer-term Emotional Impact of the Wuhan Lockdown on Wuhan and Non-Hubei Weibo Users

#### Multilevel Modeling

To examine the longer-term emotional impact of the lockdown (RQ 3), we compared the emotional trajectories in the 2 weeks after the lockdown was imposed (ie, between January 24, 2020, and February 6, 2020) of the Wuhan and non-Hubei groups. We included all 4 slopes tested in the previous model. We tested the random effect of slope 4 (ie, variability across users in the Wuhan and non-Hubei groups). Location was introduced as the level-2 predictor. We also tested the fixed effects of the remaining slopes. Multilevel model 2 is shown in Textbox 2.

**Multilevel model 2 for longer-term emotional impact.**

**Multilevel model 2**

- Level 1:
  Emotionit = *β*_0i_ + *β*_1i_ × slope 1 + *β*_2i_ × slope 2 + *β*_3i_ × slope 3 + *β*_4i_ × slope 4 + e_it_
- Level 2:
  *β*_0i_ = *γ*_00_ + *γ*_01_ × locationi + U_0i_
  *β*_1i_ = *γ*_10_
  *β*_2i_ = *γ*_20_
  *β*_3i_ = *γ*_30_
  *β*_4i_ = *γ*_40_ + *γ*_41_ × locationi + U_4i_

#### Valence

The results of multilevel model 2 with valence as the outcome variable are summarized in the upper panel of Table 3. As location was introduced as the moderator for slope 4 in multilevel model 2, the main effects of slope 4 indicated emotional trends conditional on a location of 0 (ie, the non-Hubei group). From January 24, 2020, the main effect of slope 4 for valence was 0.000 (SE 0.001; *P*=.71), indicating no valence change on average for the non-Hubei group in the 2 weeks after the lockdown was imposed. The random effect of slope 4 was significant (SD 0.021; *χ*^2^_2_=15.8; *P*<.001), indicating that the slope varied across individuals in both the Wuhan and non-Hubei groups. However, location did not moderate slope 4 (*γ*_41_=−0.004; SE 0.003; *P*=.16), indicating that, although people differed in the longer-term valence trend, the difference was not explained by the location difference between the Wuhan and non-Hubei groups.

**Table 3:** Longer-term emotional impact based on a multilevel model predicting valence or arousal with slope 4 at level 1 by location at level 2 (data set 2).

Variable			Fixed effect					Random effect				
			Coefficient		SE	*P* value	SD			Chi-square (*df*)^a^		*P* value
**Valence**												
	Intercept (*γ*_00_)	0.339		0.021		<.001	0.356		946.8 (2)		<.001	
	Slope 1 (*γ*_10_)^b^	0.124		0.013		<.001	—^c^		—		—	
	Slope 2 (*γ*_20_)^d^	0.094		0.019		<.001	—		—		—	
	Slope 3 (*γ*_30_)^e^	−0.128		0.018		<.001	—		—		—	
	Slope 4 (*γ*_40_)^f^	0.000		0.001		.71	0.021		15.8 (2)		<.001	
	Location (*γ*_01_)^g^	−0.080		0.020		<.001	—		—		—	
	Slope 4×location (*γ*_41_)^f,g^	−0.004		0.003		.16	—		—		—	
**Arousal**												
	Intercept (*γ*_00_)	2.378		0.018		<.001	0.257		500.2 (2)		<.001	
	Slope 1 (*γ*_10_)^b^	0.085		0.011		<.001	—		—		—	
	Slope 2 (*γ*_20_)^d^	0.261		0.017		<.001	—		—		—	
	Slope 3 (*γ*_30_)^e^	−0.284		0.016		<.001	—		—		—	
	Slope 4 (*γ*_40_)^f^	0.001		0.001		.56	0.016		5.9 (2)		.05	
	Location (*γ*_01_)^g^	−0.109		0.017		<.001	—		—		—	
	Slope 4×location (*γ*_41_)^f,g^	0.003		0.003		.26	—		—		—	

^a^The chi-square value is calculated as the difference in –2loglikelihood between a model with and a model without the random effect.

^b^Slope 1: emotional trend before the lockdown (between January 20, 2020, and January 22, 2020).

^c^The cells were kept empty when the random effect of a variable was not included in the model.

^d^Slope 2: difference in emotion between January 23, 2020 (the day of lockdown) and January 22, 2020.

^e^Slope 3: difference in emotion between January 23, 2020 (the day of lockdown) and January 24, 2020.

^f^Slope 4: longer-term emotional trend in the 2 weeks after the lockdown was imposed.

^g^Location: dummy coded as 1 denoting the Wuhan group and 0 denoting the non-Hubei group.

#### Arousal

The results of the multilevel model with arousal as the outcome variable are summarized in the lower panel of Table 3. From January 24, 2020, the main effect of slope 4 was 0.001 (SE 0.001; *P*=.56), indicating no significant arousal change in the non-Hubei group in the 2 weeks after the lockdown was imposed. The random effect of slope 4 was insignificant (SD 0.016; *χ*^2^_2_=5.9; *P*=.05), implying that there was not enough evidence to suggest that the slope varied across individuals in both the Wuhan and non-Hubei groups. Location did not significantly moderate slope 4 (*γ*_41_=0.003; SE 0.003; *P*=.26), indicating that there was no difference in longer-term arousal changes between the Wuhan and non-Hubei groups.

Overall, there is not enough evidence to suggest that longer-term changes in emotional valence and arousal occurred in the 2 weeks after the lockdown was imposed, regardless of geographical area.

## Discussion

### Principal Findings

Using the Wuhan lockdown as our context, we sought to understand the interplay between lockdown measures and the emotions of residents in different areas of China. Our results showed that, during the lockdown, most posts on Weibo were pleasant, with low arousal. Compared with posts before the lockdown, posts after the lockdown was imposed had higher valence and arousal, indicating more pleasant and activated emotions. Word cloud analysis revealed an increased use of the encouragement word *jiayou* after the lockdown was imposed, which might account for the increase in valence and arousal. Non-Hubei users’ valence and arousal were significantly higher on the day of lockdown than on the day before and after the lockdown was imposed. In contrast, Wuhan residents showed little immediate change in emotion in response to and after the Wuhan lockdown was imposed. Overall, in the 2 weeks after the lockdown was imposed, the valence and arousal scores of Weibo users remained constant regardless of their geographical location.

In contrast to studies that observed more negative emotions during the lockdown, our overall emotion trajectories suggest that Weibo users demonstrated more pleasant and activated emotions after the lockdown was imposed. A possible explanation for the discrepancies between our results and those of previous studies is the differences in word choice when coding the data. Studies that reported more negative emotions after the lockdown was imposed tended to use emotion types such as “stress,” “hostility,” “disappointment,” “surprise,” “fear,” “guilt,” and “blame” [2,4,25,28,32], and those that reported positive emotions after the lockdown was imposed tended to include emotion types such as “encouragement,” “admiration,” “hope,” and “blessedness” [2,28,32]. The word choices could have biased their results toward conclusions that were determined by the word categories chosen. Instead of focusing on a few selected emotion categories, we chose a lexicon based on the circumplex model of affect in which its two dimensions (valence and arousal) underlie most—if not all—emotions [34-36]. In this model, emotions are composed of valence and arousal, meaning that both pleasant and unpleasant emotions with different levels of arousal are covered.

Unlike non-Hubei people, who were significantly affected, people in Wuhan experienced smaller immediate changes in emotions in response to the lockdown event. Unlike the temporary peak in valence and arousal found in non-Hubei areas on the day of lockdown, there was a minimal change in valence and arousal for Wuhan people. This result appears to support the *psychological typhoon eye effect*, which describes the phenomenon of people closer to the epicenter of a devastating event having less intense psychological reactions to it [61]. Zhang et al [62] concluded that people closer to the outbreak displayed fewer mental health problems in response to the COVID-19 pandemic as they had a more accurate estimation of the situation and were desensitized by repeated exposure to stress.

Although the emotions of Wuhan people were more stable than those of non-Hubei residents on the day of lockdown, their emotional changes were similar to those of non-Hubei people over the longer term. Overall, Chinese Weibo users, regardless of whether they were in lockdown or not, expressed stable emotions with higher valence and arousal in the 2 weeks after the lockdown was imposed than before the lockdown. This result is in stark contrast to that of the study by Zhao et al [4], who found fewer positive emotion words (eg, “happiness”) and more negative emotion words (eg, “fear” and “pain”) in Wuhan than in non-Hubei areas after the lockdown was imposed. The difference may be rooted in the fact that, unlike our study, which selected data from up to 2 weeks after the lockdown was imposed (ie, January 23, 2020, to February 6, 2020), their data covered a longer postlockdown period (January 23, 2020, to February 16, 2020) that included the significant event of the death of Dr Li Wenliang on February 7, 2020. Li’s death was accompanied by dramatic increases in the number of posts and expression of negative emotions such as “anger,” “fear,” “surprise,” and “depression” [4,25,27], and the inclusion of this event may have negatively biased the emotional trajectories following the lockdown announcement.

We did not observe a systematic difference in valence and arousal scores between the Wuhan and non-Hubei groups after controlling for the moderating effect of location for slopes 2 and 3. That is, the average score of valence and arousal by person by day did not differ between people in or out of lockdown. This finding is different from a recent study by Meock et al [63] that compared the emotional responses to the Australian lockdown of people who were in and out of lockdown. The participants (both in and not in lockdown) in that study recorded their emotions 7 to 9 times per day for 1 week. Those in lockdown had slightly more negative emotions and slightly fewer positive emotions than those not in lockdown. A possible explanation for the discrepancy in results is the timing of data collection as Meock et al [63] collected data at a later stage of the Australian lockdown. Our study focused on the first 2 weeks of the Wuhan lockdown, during which the emotional consequences might not yet have emerged.

It is reasonable to expect negative emotions during a public health emergency such as the COVID-19 pandemic, but the power of positive emotions should not be overlooked. Our results highlight collective positive emotional responses during a public health emergency. Although Weibo users made both pleasant and unpleasant posts, their average daily emotions were pleasant and of low arousal throughout the 4 weeks under scrutiny. Unpleasant posts only accounted for 20.82% (74,796/359,190) of all posts during the lockdown. These findings are similar to those of Pan et al [64], who found that >50% of comments on Weibo during the pandemic were positive, and Arora et al [29], who found that happiness was the most prevalent emotion expressed by Indian Twitter users during the lockdown, accounting for 40% of users’ posts. Maintaining a positive emotional state may contribute to better mental health and lead to better coping when facing stressful events [62,65]. Positive feelings such as “calmness,” “hope,” “love,” and “gratitude” during the COVID-19 pandemic have been found to be positively correlated with a resilience mindset, and the relationship is stronger among people with a higher level of negative emotions (eg, “anger,” “anxiety,” “regret,” and “sadness”) than among people with low levels of negative emotions [9].

### Why Was the Lockdown Positively Received?

Why did Weibo users in China respond positively to the adverse situation after the lockdown was imposed? The cognitive processes of people in Eastern cultures are driven by Eastern naïve dialecticism, suggesting a tendency to see good in the bad and bad in the good [66,67]. Therefore, from a cross-cultural perspective, Chinese people are more likely than their Western counterparts to embrace adversity during a pandemic. Studies have found cultural differences in emotional responses during the pandemic. Yap et al [68] collected data on affect, optimism, well-being, and meaning in life from China and Canada in March 2020 and April 2020. Compared with the Canadian participants, the Chinese participants reported more positive and fewer negative emotions, greater optimism, greater psychological well-being, and greater sense of meaning during the pandemic. Our findings of higher valence and arousal after the lockdown was imposed, along with an abundance of encouragement words such as *jiayou*, add new evidence that Chinese people reacted more optimistically to the outbreak of COVID-19. Future studies could collect social media data from different countries to compare how people with different cultural backgrounds react to public health emergencies.

People tend to bond with strangers after an exogenous crisis, creating a sense of belonging to emergent groups [69,70]. Such collective cohesion and shared identity enable mutual support in adverse situations such as pandemics [71]. Research findings have also shown that a collective experience of positive emotions contributed to more resilience and better mental health during the COVID-19 pandemic [8]. Similar collective behavior was found during the Wuhan lockdown. In in-depth interviews by Qian and Hanser [21], Wuhan residents reported that they soon adapted to reality upon the abrupt announcement of the lockdown, with mutual support among neighbors on WeChat groups and in communities. Our results add further evidence for such collective behavior, with people on Weibo shouting *jiayou* together to support each other through the unfolding uncertainties of the pandemic. This finding is in line with the suggestion of Elcheroth and Drury [71] that the continuity of social ties might help people manage negative feelings during social crises such as pandemics and lockdowns.

### Implications and Limitations

Although the emotion trajectories of the Wuhan lockdown have been examined in previous studies, they have focused on limited types of emotions. Instead of coding social media data using limited types of emotions, we explored emotional responses to lockdown using a lexicon based on the valence and arousal dimensions of the circumplex model of affect [34-36]. Hence, our coding was not limited to a certain number of emotions. Rather, we sought to cover most if not all emotions.

The current study offers significant practical implications. In addition to providing an emotion map describing the valence and arousal of Weibo users, we offer explanations for the changes in emotional response patterns. We highlight the power of web-based social cohesion, which may explain the stability of Wuhan residents’ emotional states when the lockdown was imposed and the 2 weeks afterward. Such findings provide insights for policy makers to consider the reasonable length of lockdown measures.

However, our study is not without limitations. The first is the potential influence of censorship on our data sets. Posts that violate Weibo regulations are usually removed within 24 hours of their creation, meaning that real-time data scraping is crucial to understand the complete emotional picture on the web [27]. The observation that censored posts from the day of lockdown had a higher degree of criticism than of support whereas uncensored posts had similar amounts of criticism and support [27] might suggest that the real valence after the lockdown was imposed was lower than that detected in our study. To overcome this issue, future studies could consider using Weibo data collected in real time or within 24 hours after an event. Furthermore, as some posts contained words that were not coded in the CSLI lexicon, 7.38% (28,601/387,791) of the posts in data set 1 were excluded from the sentiment analysis. These posts may have contained emotion information. In the future, researchers could use machine learning and deep learning algorithms to capture the emotions embedded in the words that were not coded in the lexicon and map out emotional trajectories [2,32].

### Conclusions

In this study, we applied a circumplex model of affect on 2 data sets of Weibo posts to explore emotional responses before and after the Wuhan lockdown was imposed. Our results suggest that, even while facing the adverse situation of a lockdown, the overall aggregated emotion expressed on Weibo was pleasant and of low arousal. Over time, the web-based discussion included more encouraging messages. In addition, compared with people from more distant areas, Wuhan residents had more stable emotions on the day of lockdown, but they showed similar changes to non-Hubei residents in the 2 weeks after the lockdown was imposed. Interestingly, the lockdown did not seem to subdue the feelings of Wuhan residents despite the fact that they were physically constrained. The increased valence and arousal of posts after the lockdown was imposed might suggest a collective cohesion that provides mutual support for people under adverse situations. Policy makers could consider various approaches to forming and maintaining social connections for mutual encouragement and better adjustment during lockdowns and public health emergencies.

## Abbreviations

CSLI: Chinese Sentiment Lexicon for Internet
RQ: research question

## Data Availability

The raw data collected for this study are not publicly available as they contain Weibo users’ personal information.
